# A randomised controlled trial comparing a dietary antiplatelet, the water-soluble tomato extract Fruitflow, with 75 mg aspirin in healthy subjects

**DOI:** 10.1038/ejcn.2016.222

**Published:** 2016-11-23

**Authors:** N O'Kennedy, L Crosbie, H-J Song, X Zhang, G Horgan, A K Duttaroy

**Affiliations:** 1 Provexis Plc, London, UK; 2 Bioinformatics and Statistics Scotland (BioSS), Dundee, UK; 3 Department of Nutrition, Institute for Basic Medical Sciences, Faculty of Medicine, University of Oslo, Blindern, Oslo, Norway

## Abstract

**Background/Objectives:**

Increasing numbers of food ingredients are gaining acknowledgement, via regulated health claims, of benefits to human health. One such is a water-soluble tomato extract, Fruitflow (FF), a dietary antiplatelet. We examined relative platelet responses to FF and to 75 mg aspirin (ASA) in healthy subjects.

**Subjects/Methods:**

A total of 47 healthy subjects completed a double-blinded randomised controlled trial following a crossover design. Acute and 7-day treatments with 75 mg ASA were compared with control with and without concomitant FF, over a 5-h timecourse. Platelet aggregation response agonist, platelet thromboxane A2 release, plasma clotting times and time to form a primary haemostatic clot (PFA-100 closure time, TTC) were measured.

**Results:**

Administration of all treatments lowered platelet function and thromboxane A2 generation, and extended the TTC, relative to baseline (*P*<0.001) and to control (*P*<0.001). Plasma clotting times were not affected. A single 75 mg dose of ASA showed approximately equal efficacy to a dose of FF, whereas daily 75 mg ASA was approximately three times as effective after 7 days (*P*=0.002). Platelet responses were heterogenous with distinct weak and strong responder groups. Weak ASA responders retained a functional platelet response to collagen agonist and were responsive to FF. Concomitant FF and ASA did not lead to significant additive effects.

**Conclusions:**

The suppression of platelet function observed after consuming FF is approximately one-third that of daily 75 mg ASA. The reversible action of FF renders it less likely to overextend the time to form a primary haemostatic clot than ASA, an important safety consideration for primary prevention.

## Introduction

Several studies have shown that populations consuming a Mediterranean diet enjoy a degree of cardioprotection.^1, 2, 3^ A link to tomato consumption has been suggested.^4, 5, 6, 7^ Research has shown that the reported cardioprotective effects of tomatoes could arise in part because of antiplatelet properties of tomato fruit components.^8^ Modulation of blood clotting in this way is known to reduce the risk of thrombotic events in high-risk cardiovascular disease (CVD) patients, and may also be beneficial in primary prevention of CVD.^9, 10, 11, 12^ We have previously described a method for extracting the antiplatelet components from tomato juice, on the basis of which a new food ingredient, known as lycopene-free water-soluble tomato concentrate (Fruitflow (FF)), has been developed.^13, 14^ FF was the first ingredient to be granted a proprietary EU-wide Health Claim under Article 13.5 of the Health Claims Regulation EC 1924/2006, which was adopted in Europe in 2006. FF is now commercially produced and used in mainstream foods and food supplements with the aim of maintaining circulatory health.

A body of research work exists describing the bioactivity of FF *in vitro* and *ex vivo*^8, 13, 14, 15, 16, 17, 18, 19^ A range of human studies by ourselves and others have established that its consumption leads to a significant reduction (average 8–23%) in *ex vivo* platelet aggregation response after 3 h.^15^ A dose–response to the amount of extract consumed can be observed.^14^ The reduction in platelet function that follows a one-off dose persists for up to 18 h, and the response can be greater in men than in women.^14^ When consumed daily for at least 2 weeks, baseline platelet function is persistently lowered, and effects are no longer attenuated after 18 h.^16, 20^ The effects observed are not cumulative, that is, the extent of the reduction in platelet responsiveness to stimulus does not increase with chronic consumption. The extract contains three broad groups of bioactives, based on nucleotides, simple phenols and their conjugates, and flavonoids and their conjugates.^13, 15^ Mechanistic studies suggest that effects are mediated through effects on PPARs, prevention of p-selectin expression on the platelet surface and reduction in glycoprotein IIIbIIa.^14, 16, 17, 18, 19^

FF has been authorised for use at a daily dosage containing at least 65 mg of bioactive tomato components, associated with a mean reduction in platelet aggregability of 8–23%. However, the actual benefit of such a reduction in platelet aggregability is not easy to infer. This study set out to compare the effects of FF and 75 mg ASA in healthy subjects, in order to contextualise the effects on platelet aggregation that can be achieved by a dietary antiplatelet, and facilitate understanding of their relevance.

## Materials and methods

### Preparation and blinding of intervention supplements

FF is commercially produced by DSM Nutritional Products, Basel, Switzerland, in syrup and in powder formats. The syrup format was used in this study, at the EFSA-approved daily dose of 3 g, containing a minimum of 65 mg antiplatelet components. Aspirin (ASA; 75 mg non-coated capsules, Bayer Corporation, Whippany, NJ, USA) was obtained from a local pharmacy. FF, ASA and their corresponding controls (Treacle, Tate & Lyle, London, UK, and microcrystalline cellulose, Essential Nutrition Ltd, Brough, UK, respectively) were encapsulated using red size 00 Vegecaps (LGA, La Seyne sur Mer, France). All capsules administered were provided by Provexis plc, in sealed, desiccated containers, and were stable below 25 °C for a period of 1 week. Fresh supplement capsules were provided weekly, and coded in accordance with a randomisation protocol from Biomathematics and Statistics Scotland ((BioSS), Aberdeen, UK). All supplements were identical with respect to appearance and only differed in coding of the capsules. The treatment code of the intervention supplements was blinded for subjects, investigators and staff involved in the conduct of the study.

### Platelet aggregation measurements

Blood collection using the Monovettes system (Sarstedt, Leicester, UK), platelet-rich plasma preparation and light-transmission aggregometry were carried out as described by us previously.^14, 15^ Briefly, the platelet number in platelet-rich plasma was adjusted to 300 × 10^9^/l with autologous platelet-poor plasma. Platelet aggregation in 200 μl of the adjusted platelet-rich plasma was initiated by addition of 20 μl of adenosine diphosphate (ADP) at 3 μmol/l (Sigma Biochemicals, Poole, UK), collagen (Coll) at 2 mg/l (Horm type I, Nycomed, Munich, Germany), or arachidonic acid (AA) at 500 μmol/l (Helena Biosciences, Sunderland, UK). The progress of aggregation was monitored in an AggRam 8-channel aggregometer (Helena Biosciences, Sunderland, UK), and quantified as area under the aggregation curve (%AUC). All measurements were carried out in duplicate, within 2 h of blood sampling.

### Coagulometry

Prothrombin time (PT), thrombin clotting time and activated partial thromboplastin time estimations were performed on a CoaData 4001 coagulometer (Helena Biosciences, Sunderland, UK), as described by us previously.^14, 15^

### Measurement of thromboxane B2

Thromboxane B2, the stable metabolite of thromboxane A2 (TXA2) released from platelets during aggregation, was estimated using the Thromboxane B2 Biotrak EIA assay kit from GE Healthcare Life Sciences (Buckinghamshire, UK), after sample preparation using 100 mg C2 Amprep columns. Samples were assayed in duplicate according to the manufacturer's instructions.

### Measurement of time to form a primary haemostatic clot in whole blood

The time to primary clot formation (time to clot, TTC) was measured in whole blood withdrawn into acid citrate dextrose anticoagulant, using the Platelet Function Analyzer (PFA)-100 System (Siemens Healthcare, Malvern, PA, USA). Within 90 min after blood sampling, the closure time of the PFA-100 was recorded using the manufacturer's operating protocol with Coll-epinephrine cartridges. This measurement estimates the overall function of primary haemostasis.

### Supplementary measurements

After each withdrawal of blood, full blood counts were performed using a Sysmex haematology analyzer (Sysmex UK Ltd, Milton Keynes, UK), and plasma fibrinopeptide A concentration was measured by competitive ELISA (Zymutest fibrinopeptide A assay, HYPHEN BioMed, France), as previously described.^14, 15^ Baseline plasma C-reactive protein concentration was measured in EDTA-anticoagulated blood using a semi-quantitative latex agglutination assay (Dade Behring, Milton Keynes, UK), which allowed classification of sample C-reactive protein status as either ‘normal' or ‘elevated' (>6 ng/ml C-reactive protein). Baseline fasting plasma lipid and plasma glucose concentrations were measured by a colorimetric autoanalyser method utilising specific autoanalyser analysis kits (KONE Instruments autoanalyser, Labmedics Ltd, Abingdon On Thames, UK).

### Subjects

**Figure 1:**
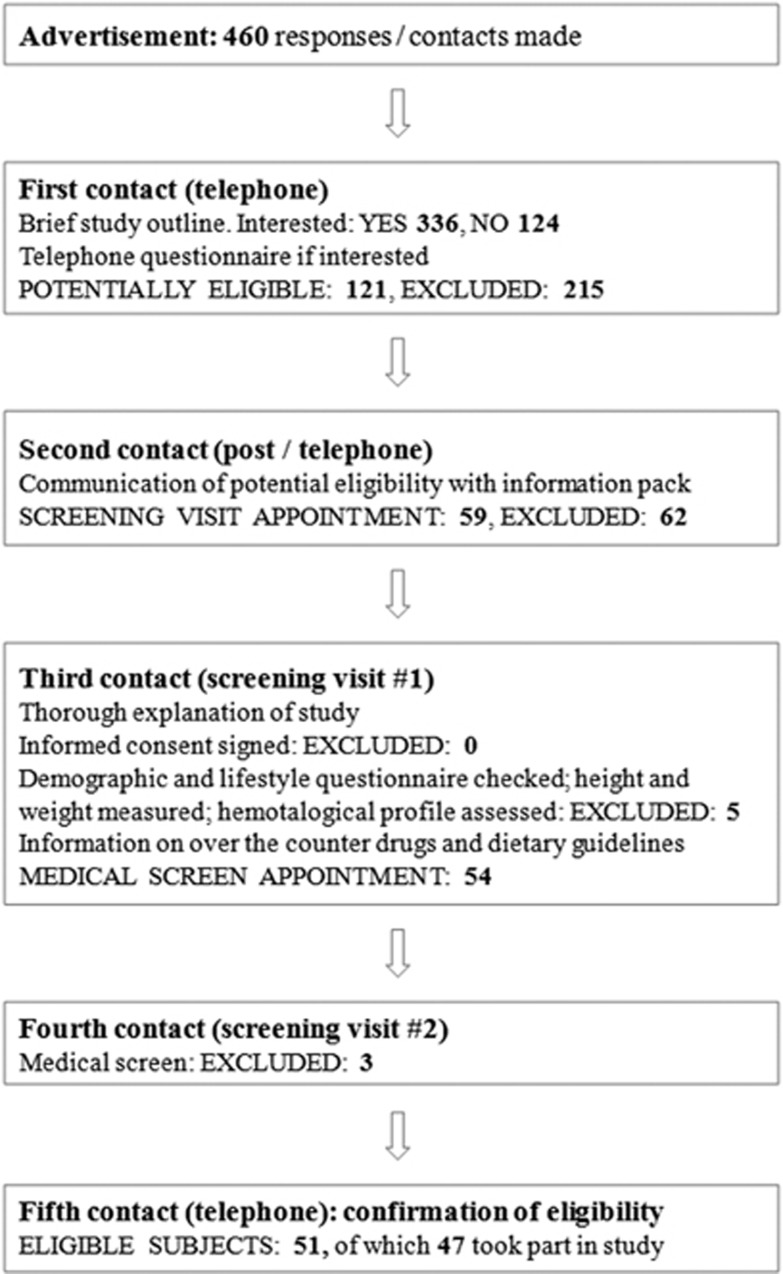
Study recruitment procedure and disposition of subjects resulting in recruitment of 47 eligible subjects.

A total of 47 healthy adults of both sexes were recruited into the study, on the basis of a 90% power calculation with a 95% confidence interval for the mean and allowing for a 10–15% drop-out rate. An outline of the recruitment procedure and the disposition of subjects is shown in Figure 1. Subjects were aged 45–75 years, with no medical history of serious disease, haemostatic disorders or intolerance of aspirin. Suitability for inclusion in the study was assessed by diet and lifestyle questionnaires (included as Supplementary Information) and medical screening, during which platelet function and coagulation was assessed. Individuals with low haematology counts (platelet number <170 × 10^9^/l; haematocrit <40% for males or <30% for females; haemoglobin <120 g/l for males or <110 g/l for females), low platelet function (as determined by response to 3 μmol/l ADP agonist) or PT values outside the normal range of 10–16 s were not included in the study. Any subject habitually consuming dietary supplements (for example, fish oils, evening primrose oil) suspended these supplements for a minimum period of 1 month before participating in the study. Subjects were instructed to abstain from consuming drugs known to affect platelet function for a 10-day period prior to participation. Written informed consent was obtained from all subjects prior to participation. All study procedures were in accordance with the Helsinki Declaration of 1975 (revised in 1983), and the study was approved by the North of Scotland Research Ethics Committee (reference 08_S0802_196). This study was registered at www.controlled-trials.com as ISRCTN84856452.

### Randomisation

Eligible subjects were randomised using computer-generated random digit selection (provided by BioSS), based on entry in the study.

### Study design

**Figure 2:**
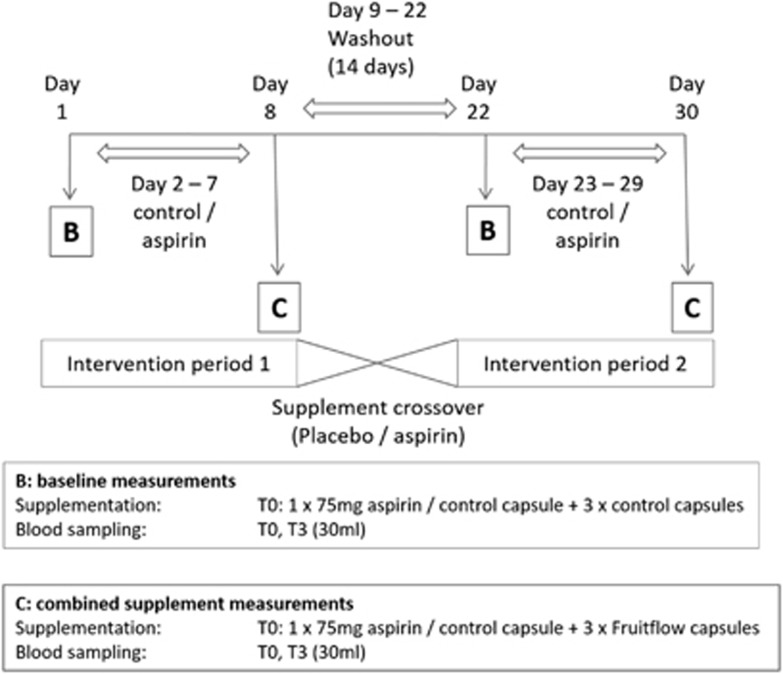
Study design, treatment schedule and blood sampling schedule. Subjects attended the study centre in a fasting state on 4 occasions (Days 1, 8, 22 and 30). On day 1, the subjects provided an initial baseline blood sample (T0) of ~30 ml, before consuming one 75 mg ASA capsule along with 3 placebo capsules (ASA1), or an identical control (Con1). After 3 h (T3), a second blood sample was taken after which the subjects received a low GI snack (breakfast bar) with some water. After the final blood sample was taken, the subjects were offered lunch and were free to depart. During the 6 days (days 2 to 7) following their first test day, the subjects were placed on a regimen of one 75 mg ASA (or identical placebo) capsule once a day (as per their day 1 treatment allocation), to be taken before 9am with food. On the morning of day 8, the subjects returned to the study centre in a fasted state. After the initial baseline blood sample (at T0), the subjects consumed one 75 mg ASA capsule or identical placebo (as per their day 1 treatment allocation) along with 3 capsules of FF, a dose of at least 65 mg tomato-derived antiplatelet components. After the T3 blood sample was taken, the subjects were offered lunch and departed. A minimum 14-day washout period then followed, after which the subjects returned to complete the study according to their crossover schedule.

The study followed a randomised double-blinded placebo-controlled crossover design with two 8-day intervention periods, separated by a minimum 14-day washout period (~30 days in total). All study activities were carried out in the Human Nutrition Unit of the University of Aberdeen Rowett Institute of Nutrition and Health, Aberdeen, UK. The primary outcomes were post-supplement changes in platelet aggregation response to agonist, changes in TXA2 generation by platelets and changes in TTC. Secondary outcomes included post-supplement changes in plasma clotting times. Figure 2 shows the outline of the study, and the intervention pattern on study days. To prevent activation of the haemostatic system, the maximum number of attempts at venepuncture was limited to four per visit. As in our previous studies, any blood sample showing evidence of activation, defined as an fibrinopeptide A concentration higher than 7 μg/l, was discarded. Any subject showing an elevated inflammatory response, evidenced by a baseline C-reactive protein concentration higher than 6 mg/l, was withdrawn from the study for the period affected, and the scheduled intervention was undertaken at a later date.

### Statistics

Data are presented as mean±s.d. Data from interventions with fibrinopeptide A above 7 μg/l were removed from the set. This resulted in loss of 134 of the 564 data points collected (24%), distributed between the four treatments—ASA1 (38, 5%) representing one 75 mg ASA dose, ASA7 (33, 6%) representing a 7-day 75 mg ASA dose, Con1 (28, 7%) representing one control supplement and FF1 (33, 6%) representing one FF dose. Preliminary assessment of the data distribution was carried out by inspecting histograms, and data points classified as outliers were removed. Changes from baseline (T0) within the study population were analysed using a mixed model following the residual maximum likelihood (REML) approach. Variances were similar between comparison groups. Initially, random effect terms were subject/(visit x time point), whereas fixed effect terms were (order+treatment+gender) x time point. Significance was tested with the Wald statistic. *Post hoc* multiple comparisons were carried out using a two-tailed Student's *t*-test. As no significant order x treatment or gender x treatment interactions were observed, the model was simplified (without the order term, treatment and visit are equivalent). Random effects then became subject/(treatment x time point), and fixed effects were treatment x time point.

Age, smoking behaviour, body mass index, habitual tomato consumption, baseline fasting plasma lipid concentration and plasma glucose concentration were included as potential confounding factors in all analyses, but none of these parameters had any significant effect on the results of any of the measurements. Exploratory analysis was used to test for associations between variables using the Pearson correlation coefficient. All statistical analyses were carried out using Genstat (VSN International, 17th/18th edition), and differences were considered significant at *P*<0.05.

## Results

### Baseline characteristics of subject group

The baseline characteristics of the 47 study subjects are given in Table 1. Baseline platelet response to agonist was high, judged from high responses to 3 μmol/l ADP and 2 mg/l collagen, compared with those for 7.5 μmol/l ADP and 3 mg/l collagen. This indicated that for most subjects, using the higher agonist concentrations in aggregation studies would result in overstimulation. Therefore results following are presented for 3μmol/l ADP and 2 mg/l collagen in all cases. This allows comparison with previous studies conducted by us, in which 3 μmol/l ADP was similarly used.^14, 15^

The extrinsic and intrinsic coagulation pathways, as represented by PT/TCT and aPTT, respectively, appeared normal, as were the TTC times which fell within the normal range for the C/E cartridges.

### A single dose of 75 mg ASA and FF had similar effects on overall haemostatic function

Table 2 compares the haemostatic effects of a single dose of ASA or FF to control, 3 h after consumption. Control supplementation did not alter any haemostatic marker measured from its baseline values (Table 2, treatment Con1). A single dose of ASA (ASA1) and a single dose of FF (FF1) both significantly reduced the platelet aggregation response to agonist, relative to baseline and to control. ASA1 was significantly more effective in reducing arachidonic acid- and collagen-mediated aggregation than FF1 (*P*<0.001, *P*=0.028 respectively). The associated reductions in TXA2 formation were also significant for both treatments. For ADP-mediated aggregation, there was no difference between ASA1 and FF1 treatments (*P*=0.733), both reducing ADP-mediated aggregation by ~26% relative to baseline. No effects on coagulation system markers (plasma clotting times) were observed for either treatment but the reductions in platelet aggregatory potential achieved nevertheless led to a significant increase in TTC, the time to form a primary haemostatic clot in the PFA-100 analyser. The overall increases in TTC observed did not differ significantly between the two treatments (*P*=0.473). Thus the larger effects on both arachidonic acid- and collagen-mediated platelet aggregation observed after a single dose of ASA did not translate into a significantly larger effect on primary haemostasis overall.

### Daily 75 mg ASA for 7 days completely inhibited the platelet response to AA and associated TXA2 production but did not fully inhibit the platelet collagen or ADP responses

Subjects consumed a single daily dose of either 75 mg aspirin or control before 0900 hours for 7 days. Seven days of control supplementation did not significantly alter any of the variables measured from study baseline values (Table 2, treatment Con7). Seven days of 75 mg aspirin supplementation (treatment ASA7) significantly altered all platelet function parameters, and the TTC, compared with both baseline and control, without increasing plasma clotting times from either baseline or control. Extending supplementation to 7 days did not significantly increase the inhibition of either ADP- or collagen-mediated aggregation from that achieved with a single dose of ASA (*P*=0.063, *P*=0.135 respectively). However, inhibition of arachidonic acid-mediated aggregation was close to total, generation of TXA2 was reduced by over 90%, and the TTC after 7 days of ASA was significantly longer than that after a single dose. In terms of overall primary haemostasis, 7 days' of 75 mg ASA treatment had approximately three times greater effect than a single dose of FF.

### Combining dietary FF with daily 75 mg ASA usage did not lead to any clinically dangerous additive effect

Combining one dose of FF with daily 75 mg ASA resulted in small additive effects, notably in terms of further reductions in ADP- and collagen-mediated aggregation, compared with daily ASA alone (Table 2, treatment ASA7+FF). However, these effects were not significant, either statistically or clinically, as evidenced by the lack of changes in overall TTC from the values measured for daily ASA alone.

### The haemostatic responses of the study population were markedly heterogenous for all treatments

**Figure 3:**
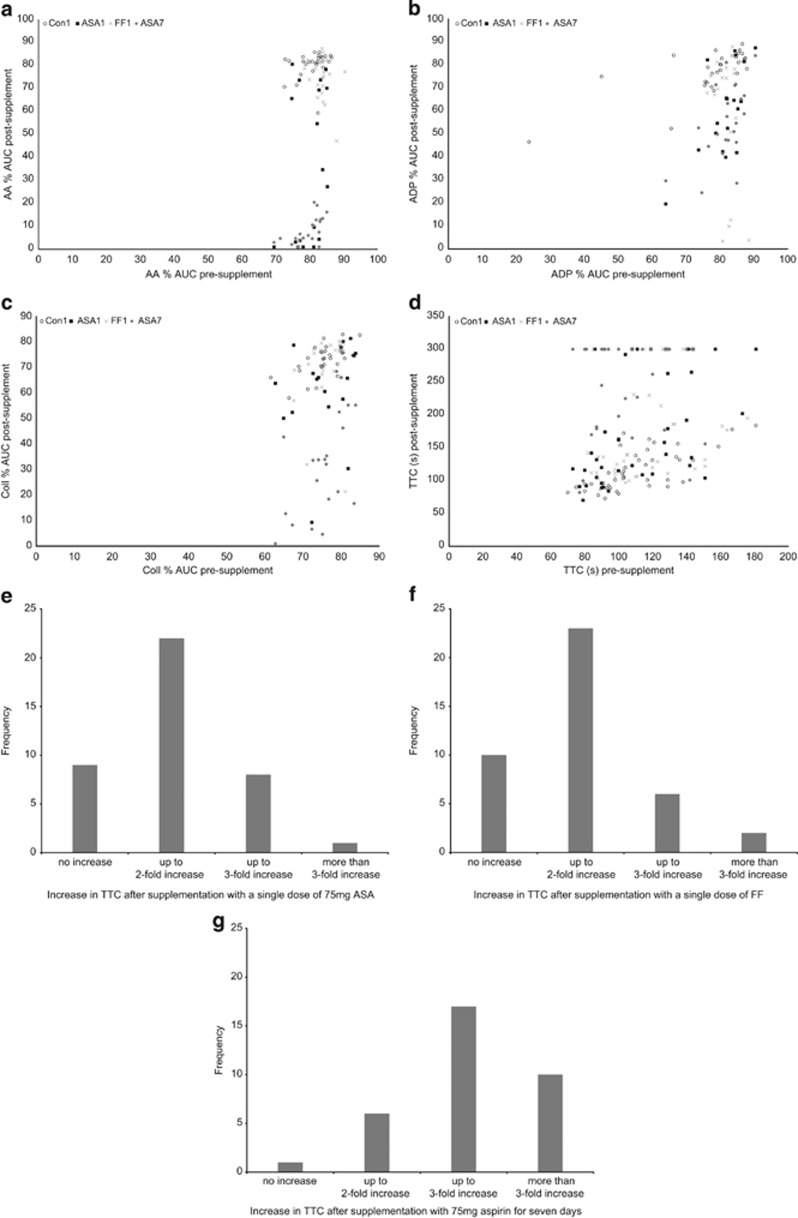
Heterogeneity in response to treatments observed in the subject group. (**a**–**d**) Scatterplots showing the wide range observed for the inhibition of AA-mediated aggregation (**a**), ADP-mediated aggregation (**b**), collagen-mediated aggregation (**c**) and TTC (**d**). T0 values are plotted on the *x*-axis, whereas the corresponding T3 values are plotted on the *y*-axis, for all subjects for whom pairs of values were obtained. (**e**–**g**) Histograms of the TTC data for each of the three treatments FF (**e**), ASA1 (**f**) and ASA7 (**g**), illustrating the spread of the data and the patterns of the inhibition of TTC observed.

Figures 3a–d illustrates the range of responses to the three intervention supplements in this group of subjects. Data are plotted for each platelet aggregation pathway (a–c), and for the overall TTC (d). The plots are suggestive of different patterns of response within the overall subject group. Although such patterns might have been expected for individual platelet pathways, it was interesting to see that the heterogeneity remained in the TTC data. Figures 3e–g summarise the TTC data in histogram format. FF1 and ASA1 treatments gave very similar patterns of response, with the majority of the study population experiencing a moderate increase in TTC (up to twofold increase) after supplementation, and a small group responding more strongly. The opposite was true for ASA7 treatment, where the majority experienced a lengthening of the TTC by more than twofold baseline values. For 29% of the study population, the TTC increased by more than threefold after ASA7.

In order to examine the platelet behaviour behind the increases in TTC, the study subjects were classified into groups. Non-responders to treatment were defined as those experiencing no increase in TTC after supplementation. The remaining ‘responders' were divided into two groups, those showing up to twofold increase in TTC after treatment (‘moderate' responders), and those showing greater than twofold increase in TTC after treatment (‘high' responders). The platelet characteristics of the responses to treatment for each of these groups were then examined (Table 3).

### Study subgroups with differing increases in TTC may have different underlying platelet responses to both FF and ASA

Within the ‘moderate' and ‘high' responder categories, the average increases in TTC were remarkably consistent across treatments, with moderate responders averaging 30–40% increases in TTC, and high responders averaging 154–170%, irrespective of the supplements given. High FF responders showed very strong inhibition of 3μmol/l ADP-related platelet aggregation, more than three times greater than the majority of moderate FF responders. High ASA1 responders did not share this characteristic, having instead a markedly greater inhibition of collagen-mediated platelet aggregation than the majority of moderate ASA1 responders. Subjects responding moderately to ASA7 did not show differences from high responders in either arachidonic acid- or ADP-mediated aggregation, but the inhibition of collagen-mediated aggregation was half of that seen for high ASA7 responders. This suggests that a high response to FF may be experienced if the subject is more than normally sensitive to blockade of ADP-mediated aggregation, whereas a high response to ASA seems to be associated with successful disruption of collagen-mediated aggregation, rather than arachidonic acid-mediated aggregation.

## Discussion

In this study, we set out to examine the broader relevance of the reduction in platelet function observed after consumption of FF in a dose of at least 65 mg bioactive tomato compounds. We sought to establish this by carrying out a crossover within-subject comparison of FF and 75 mg ASA, in healthy subjects fitting the target population for FF consumers. The health outcomes associated with 75 mg ASA consumption are established, and by quantifying its effects on platelets in this study population we hoped to put the corresponding effects of FF into a more clinical context. We also wished to assess any potential interactions which might arise, should FF and daily ASA be combined.

To combine these aims, we designed a study in which subjects consumed either control supplements or 75 mg ASA daily for 7 days. On the first day of each treatment, we measured the effect of a single supplement (control or ASA). On the seventh day of each treatment, we measured the effect of daily supplement (baseline sample – control or ASA), and then combined each treatment with FF. This allowed us to measure the combinations of daily ASA+FF, and control+FF, which we treated as equivalent to a single dose of FF. The effects of a single dose of FF taken after 7 days of control supplementation were compared to earlier results for a single dose of FF taken alone, and were found to be very similar (26% reduction in ADP-mediated aggregation vs 20/21% in previous studies;^14, 15^ 10% reduction in collagen-mediated aggregation versus 15% in a previous study^15^), improving confidence that the control+FF measurement could be regarded as representing the effect of a single dose of FF. Because of the slightly unbalanced design, the study results must be interpreted with a degree of caution, but the comparisons made between treatments appear fit for purpose.

We first compared the response of individual platelet aggregation pathways with FF and ASA, to observe similarities and differences in mode and timescale of action. We then examined treatment effects on a marker of overall platelet aggregation (released TXA2), so that the platelet-specific component of the treatment effects could be compared without considering exact mechanisms. Finally, we quantified and compared the effects of ASA and FF treatments on time to form a primary haemostatic clot, to allow the practical relevance of observed antiplatelet activities on the wider haemostatic system to be assessed.

Results showed that within subjects, differing effects of 75 mg aspirin and FF on individual platelet aggregation pathways united to extend the time taken to form a primary clot by 57% after a single dose of 75 mg ASA, and by 48% after a single dose of FF. Thus a single dose of ASA was about as effective in suppressing primary haemostasis as a single dose of FF, in this study population. For both treatments, this was achieved entirely via platelet suppression, as plasma clotting times were unaffected. When 75 mg ASA was consumed daily for at least 7 days, we found the difference in efficacy between ASA and FF to increase so that daily ASA was about three times as effective in suppressing primary haemostasis as a single dose of FF. As differences in acute effects may well arise for metabolic as well as mechanistic reasons, we feel that the comparison with daily 75 mg ASA usage, when acute metabolic factors are less important, gives the most physiologically relevant estimate of FF potency.

Our conclusions do not imply that consuming three times as much FF would achieve the same efficacy as daily ASA. Previous studies have shown that consuming more than 65 mg tomato bioactive compounds per dose does not result in significantly greater inhibition of platelet function;^14^ the effects of FF on platelets, unlike those of ASA, are reversible and non-cumulative—a very important distinction between the treatments.^15^ The cumulative actions of daily ASA led to a greater than threefold increase in TTC for 29% of the study population. The internal bleeding associated with such increases contraindicates ASA for use in primary prevention.^21, 22^ A dietary antiplatelet with approximately one-third the antiplatelet efficacy of ASA that suppresses primary haemostasis by an average 47% rather than by an average 128% may be suitable for primary prevention when daily dosing with 75 mg ASA is not.

No clinically relevant interactions between daily 75 mg ASA and FF were observed in this study. That is, the combination of both treatments tested does not appear to be dangerous in terms of increasing the TTC in a clinically relevant manner. Some increases in platelet suppression were observed, mainly affecting the ADP pathway, where FF has higher potency, but the increases were not physiologically or clinically relevant overall. This conclusion applies to the population studied, and may not remain true in all contexts.

Our study has highlighted the fact that diverse effects of both ASA and FF treatments may be expected between individuals. Our study population did not appear to contain any truly ASA-‘resistant' subjects, as evidenced by the consistent reduction of AA-mediated aggregation and TXA2 synthesis after all ASA treatments.^23^ However, 53% of the study group responded less than the average to a single dose of ASA, and 19% of the study population responded significantly less than the average to 7-day ASA treatment. In this moderate ASA responder subgroup, residual ADP- and collagen-induced platelet aggregatory capacity remained higher than the overall study group average. It is possible therefore that this population subgroup has a higher than average platelet response to ADP and collagen signalling, which to some degree circumvents the action of ASA. In a clinical setting, this type of problem is overcome by using dual antiplatelet therapy rather than using single therapies. In a primary prevention setting, the advantages of a dietary antiplatelet with more than one mechanism of action, such as FF, are analogous.

Given the very heterogenous nature of baseline platelet function, the complexities of predicting which subjects may respond better to one antiplatelet over another are clear. In this study group, it appeared that high responders to FF showed greater suppression of the platelet ADP–signalling pathway than high ASA responders did. A high ASA response seemed to be linked to strong suppression of collagen-mediated signalling. However, the study group was small and composed of healthy subjects, and these observations may not apply in other populations.

To conclude, our study has shown that FF, taken in the EFSA-approved daily dosage which supplies at least 65 mg tomato bioactive components, shows approximately one-third the antiplatelet efficacy of daily 75 mg aspirin. Although a small number of subjects showed a high response to FF, with antiplatelet efficacy similar to that seen for ASA, the majority of the study population experienced moderate platelet suppression. The level of platelet suppression achieved, and the reversibility of its antiplatelet effects, suggest that FF may be appropriate for use as a dietary antiplatelet.

## Figures and Tables

**Table 1:** Descriptive statistics of demographics and baseline parameters relating to blood chemistry, platelet function, coagulation and TTC of the study population

	*Variable*	n	*Mean*	*S.e.m.*	*Min*	*Max*	*Missing values*
Demographics of population at baseline	Age (years)	47	60.54	7.15	45	72	0
	BMI	47	26.62	3.54	20.04	35.75	0
	Tomato intake (g/week)	45	960.7	622.2	0	3180	2
Blood chemistry of population at baseline (mg/l in fasted plasma)	Cholesterol	47	5.258	0.836	3.248	6.984	0
	HDL	47	1.238	0.466	0.439	2.498	0
	LDL	46	3.507	0.773	1.288	5.14	1
	Triglycerides	47	1.155	0.555	0.341	2.806	0
	Chol:HDL ratio	46	4.84	1.696	1.799	9.941	1
	Glucose	47	5.194	0.768	3.887	7.997	0
Platelet aggregation behaviour of population at baseline (%AUC)	ADP (3 μmol/l)	32	79.75	12.86	23.75	90.48	15
	ADP (7.5 μmol/l)	32	84.16	4.17	72.28	91.01	15
	Collagen (2 mg/l)	32	74.85	5.17	61.52	81.84	15
	Collagen (3 mg/l)	32	77.43	4.42	65.09	84.56	15
	AA (500 μmol/l)	31	80.21	4.02	71.25	86.4	16
TXB2 generation in baseline samples (mg/l platelet-rich plasma)	Collagen (2 mg/l)	32	172.6	62.7	64.7	347.2	15
	AA (500 μmol/l)	31	731.2	171.8	48.1	891.8	16
Plasma clotting times of population at baseline (s)	PT	32	15.22	1.40	12.5	18.7	15
	TCT	34	16.94	1.60	14	20.7	13
	aPTT	34	34.19	4.60	27	44.5	13
PFA-100 closure times of population at baseline (s)	C/E	42	116.2	29.16	73	181	7

Abbreviations: AA, arachidonic acid; ADP, adenosine diphosphate; aPTT, activated partial thromboplastin time; AUC, area under curve; BMI, body mass index; C/E, Coll-epinephrine; HDL, high-density lipoprotein; LDL, low-density lipoprotein; PT, prothrombin time; TCT, thrombin clotting time; TTC, time to clot.

Of the 47 subjects recruited, 20 were male and 27 female. None were smokers. None of the baseline parameters measured showed significant interactions with gender, age, BMI, tomato intake or plasma lipid levels.

**Table 2:** Haemostatic system variables measured before and after treatment with 75 mg ASA (single dose, ASA1 and 7-day dose, ASA7), FF (single dose, FF1) or control (single dose, Con1 and 7-day dose, Con7) supplements.

		*Baseline*^a,b^	*Acute treatments*			*7-day treatments*		*Concomitant ASA + FF*
			*Con1*^c^	*ASA1*^c^	*FF1*^d^	*Con7* e	*ASA7* e	*ASA7+FF* f
Platelet aggregation in response to agonist (%AUC)	AA (500 μmol/l)	80.4±4.1	80.4±2.7 (−0.6±5.6 %)	33.2±2.8 (−56.5±6.4%)^*,**^	70.9±2.8 (−15.8±6.0%)^*,**^	81.6±2.6 (0.7±5.2%)	9.3±2.7 (−88.5±6.1%)^*,***^	7.4±2.8 (−17.8±6.0%)
	ADP (3 μmol/l)	79.1±6.0	76.1±2.9 (0.77±4.8%)	60.6±3.0 (−26.7±5.9%)^*,**^	59.3±3.0 (−26.3±5.3%)^*,**^	81.1±2.8 (4.0±5.6%)	57.4±2.9 (−26.3±5.9%)^*,***^	50.9±3.0 (−11.7±5.3%)
	Collagen (2 mg/l)	75.6±5.7	73.1±2.4 (−3.1±3.9%)	60.4±2.5 (−23.5±4.6%)^*,**^	67.6±3.0 (−9.7±4.1%)^*,**^	74.8±2.3 (−0.9±4.6%)	37.9±2.4 (−50.0±4.5%)^*,***^	26.1±2.4 (−32.9±4.2%)
TxB2 generation induced by aggregation in response to agonist (ng/ml)	AA (TxB2)	770.3±84.7	756.1±30.7 (−1.7±6.5%)	235.6±32.7 (−62.8±7.2%)^*,**^	515.5±32.3 (−39.8±6.8%)^*,**^	782.9±29.6 (2.0±6.8%)	7.7±31.0 (−98.9±6.8%)^*,***^	5.5±31.8 (−16.0±6.9%)
	Collagen (TxB2)	194.1±50.2	180.1±8.9 (−2.2±5.8%)	53.0±9.3 (−69.3±6.8%)^*,**^	126.8±9.2 (−34.1±6.3%)^*,**^	195.6±8.5 (3.7±6.3%)	16.2±8.9 (−91.4±6.3%)^*,***^	9.9±9.1 (−43.0±6.3%)
PFA-100 closure time (s)	C/E cartridge	110.1±23.1	116.4±8.8 (5.9±8.4%)	178.4±8.9 (56.7±8.4%)^*,**^	164.4±8.9 (47.6±8.4%)^*,**^	114.4±8.8 (2.1±8.4%)	255.1±9.5 (127.8±8.4%)^*,***^	258.2±10.3 (8.6±10.4%)
Plasma clotting times (s)	PT	14.6±1.3	14.4±0.2 (−0.8±1.7%)	14.7±0.3 (−3.5±1.9%)	14.7±0.3 (1.1±1.9%)	14.4±0.2 (−1.4±1.9%)	14.4±0.2 (−1.2±1.7%)	(14.2±0.3) (−0.8±1.8%)
	TCT	16.5±1.6	16.5±0.3 (−1.3±1.1%)	16.8±0.3 (−1.3±1.2%)	16.9±0.3 (1.1±1.1%)	16.6±0.3 (−0.8±1.2%)	16.9±0.2 (0.7±1.1%)	16.7±0.3 (−0.5±1.2%)
	aPTT	33.5±4.2	34.0±0.6 (0.0±1.2%)	34.2±0.6 (1.4±1.4%)	34.1±0.6 (1.7±1.3%)	33.6±0.6 (−1.6±1.2)	33.8±0.6 (−0.9±1.3%)	33.9±0.6 (1.2±1.3%)

Abbreviations: AA, arachidonic acid; ADP, adenosine diphosphate; aPTT, activated partial thromboplastin time; AUC, area under curve; C/E, Coll-epinephrine; PT, prothrombin time; TCT, thrombin clotting time.

a Mean±s.d., with % change from baseline data in parentheses (all such values).

b Baseline denotes the overall study baseline, that is, visit 1, t0 for all subjects. This overall study baseline was not used to calculate all the % change from baseline data shown, see following footnotes.

c For the acute treatments Con1 and ASA1, % change is calculated using the formula % change from baseline=((t3−t0)/t0)%, where t0 and t3 both derive from either visit 1 or visit 3, depending on treatment order.

d For acute treatment FF1, % change is calculated using the formula % change from baseline=((t3−t0)/t0)% where t0 and t3 both derive from either visit 2 or visit 4, and when the 7-day treatment preceding this visit was the control treatment.

e For 7-day treatments Con7 and ASA7, % change refers to % change from t0 on treatment day 1, that is, ((t0 treatment day 7–t0 treatment day 1)/t0 day 1)%, where day 1 and day 7 refer to either visit 1 and visit 2, or visit 3 and visit 4, respectively, depending on treatment order.

f For concomitant treatment with ASA7 and FF, % change refers to % change from ASA7, that is, ((t3 treatment day 7–t0 treatment day 7)/t0 treatment day 7)%, where the treatment given was ASA for 7 days, and the data were collected on either visit 2 or visit 4 depending on treatment order.

*Significantly different from study baseline values, *P*<0.001 (REML followed by *post hoc* student's *t*-tests).

**Significantly different from study treatment Con1, *P*<0.001 (REML followed by *post hoc* student's *t*-tests).

***Significantly different from study treatment Con7, *P*<0.001 (REML followed by *post hoc* student's *t*-tests).

**Table 3:** Responder subgroups for each of the study treatments ASA1, ASA7 and FF1

	*Proportion of study group (%)*	*Changes from baseline haemostatic marker values (%)*					
		*TTC*	*Platelet aggregation response*			*Post-aggregation TxB2 generation*	
			*AA*	*ADP*	*Coll*	*AA*	*Coll*
*Responder subgroup with up to twofold increase in TTC*							
FF	56	31.2±24.1	-8.5±8.1	−21.6±32.3	−11.6±5.7	−31.0±36.0	−34.4±39.8
ASA1	55	42.3±27.5	−40.5±39.6	−22.9±27.4	−5.2±12.9	−64.4±27.0	−56.5±9.4
ASA7	18	37.8±35.1	−95.3±2.0	−30.7±18.6	−23.3±9.0	−96.6±1.4	−87.3±2.4
*Responder subgroup with more than twofold increase in TTC*							
FF	15	165.0±61.6	−28.7±12.2	−65.8±34.7	−35.5±35.2	−76.7±27.5	−87.9±7.8
ASA1	20	154.3±46.4	−78.3±37.7	−30.6±13.0	−42.0±31.9	−81.0±34.6	−82.8±13.8
ASA7	79	169.8±61.6	−88.9±7.4	−34.7±17.6	−55.5±31.9	−96.2±2.0	−88.4±13.2

Abbreviations: AA, arachidonic acid; ASA1, single dose of ASA; ASA7, 7-day 75 mg ASA; Coll, collagen; FF, Fruitflow; TTC, time to clot.

Within each treatment, responders were defined as those subjects showing a lengthening of the TTC, compared with baseline values. Moderate responders were defined as responders with a TTC increase of up to twofold, compared with baseline values, whereas high responders were defined as responders with a greater than twofold increase in TTC.

Mean±s.d. (all such values).

## Supplementary Material

Supplementary Information_study screening part 1

Supplementary Information_study screening part 2

Supplementary Information_study screening part 3

Supplementary Information_study screening part 4
