# Supplementary material for: A randomised controlled trial comparing a dietary antiplatelet, the water-soluble tomato extract Fruitflow, with 75 mg aspirin in healthy subjects
Source: Eur J Clin Nutr. 2016 Nov 23;71(6):723–30. doi: 10.1038/ejcn.2016.222 (PMC5470100; doi:10.1038/ejcn.2016.222)
Supplement: Supplementary Information_study screening part 1 [file ejcn2016222x1.doc]

Telephone Questionnaire (Confidential)

**SUBJECT NUMBER:**

| 1. Have you had any serious illnesses or operations in the past? |
| --- |
| 1. Do you have any chronic illnesses (e.g. diabetes, heart disease, thyroid disorder or GI irritation)? |
| 1. Are you currently taking any medication (incl. aspirin & other anti-inflammatories)? |
| 1. Are you currently taking any evening primrose oil or fish oil supplements? |
| 1. Are you currently taking any other supplements or herbal remedies? |
| 1. Do you have any allergies, including to tomatoes & aspirin? |
| 1. Is there any other reason why you should not take aspirin, to your knowledge (e.g. doesn’t agree with you) ? |
| 1. Are you a non smoker? |

Comment:

Investigator: Date:

Screening Checklist (Confidential):

**SUBJECT NUMBER:**

| 1. Weight:   Height:  BMI:  BMI between18 and 38 kg/m^2^: | Yes/ No |
| --- | --- |
| 1. Platelet number above 170 x 10^9^/ L: | Yes/ No |
| 1. Veins suitable for blood sampling and/or cannulation: | Yes/ No |
| 1. Haematocrit above 40% for males and above 35% for females: | Yes/ No |
| 1. Haemoglobin above 120 g/L for males and 110 g/L for females: | Yes/ No |
| 1. Has subject given a pint of blood for transfusion purposes in the past month before entry into the study?     Date of last blood donation: | Yes/ No |
| 1. Is subject known or to be pregnant, or does subject suspect they could be pregnant? | Yes*/ No / Not applicable  * If yes, exclude. |

***Attach whole blood count:***

***To be completed by medical staff approved by University of Aberdeen Rowett Institute of Nutrition and Health prior to subject participation***

Is the subject eligible to participate in the study?

□ NO Do not enter subject into study.

□ YES Proceed with protocol

Comments:

Investigator’s Signature: Date:

TO BE KEPT WITH LIFESTYLE QUESTIONNAIRE
