# Supplementary material for: A randomised controlled trial comparing a dietary antiplatelet, the water-soluble tomato extract Fruitflow, with 75 mg aspirin in healthy subjects
Source: Eur J Clin Nutr. 2016 Nov 23;71(6):723–30. doi: 10.1038/ejcn.2016.222 (PMC5470100; doi:10.1038/ejcn.2016.222)
Supplement: Supplementary Information_study screening part 2 [file ejcn2016222x2.doc]

*This study is funded by Provexis Natural Products Limited (*[*www.provexis.com*](http://www.provexis.com/)*), and run in association with The University of Aberdeen Rowett Institute of Nutrition and Health*

Personal Details (Confidential)

Name: ……………………………….

Address (including postcode):

……

……………………………………………………………………………………………………………….......

……………………………………………………………………………………………………………...........

Telephone (Home): ..............................................................................................................................................

Telephone (Work): ………………………………………………………………………………………….......

Telephone (Mobile): …………….........................................................................................................................

Date of Birth: ........................................................... Age: ……....………………………………….................

Occupation: ……………………………………………………………………………………………………..
