# Supplementary material for: A randomised controlled trial comparing a dietary antiplatelet, the water-soluble tomato extract Fruitflow, with 75 mg aspirin in healthy subjects
Source: Eur J Clin Nutr. 2016 Nov 23;71(6):723–30. doi: 10.1038/ejcn.2016.222 (PMC5470100; doi:10.1038/ejcn.2016.222)
Supplement: Supplementary Information_study screening part 3 [file ejcn2016222x3.doc]

*This study is funded by Provexis Natural Products Limited (*[*www.provexis.com*](http://www.provexis.com/)*), and run in association with The University of Aberdeen Rowett Institute of Nutrition and Health*

Volunteer Questionnaire (Confidential)

**SUBJECT NUMBER:**

*Please could you provide us with the following information:*

Your Height: ................................................................... Weight: ..................................................................

Has your weight been stable during your adult years? - give details ………………………………………….

Do you smoke?................................. If yes, how many a day?

If stopped smoking, when did you stop?

What regular exercise do you do and how often is this?

Do you eat any particular diet? (e.g. vegetarian, gluten free, weight loss incl. allergies)

Do you take any supplements? (e.g. vitamin tablets, herbal supplements, fish oil)

……..

………………………………………………………………………………………………………………......

What is your alcohol consumption (number of drinks per week)?

What foods will you not eat?

Do you have any allergies, including to tomatoes & aspirin?

…………………………………………………………………………………………………………..............

Do you tolerate taking aspirin well?.....................................................................................................................

Do you have any chronic illness? (e.g. diabetes, heart disease, thyroid disorder, gastrointestinal problems)

…………………………………………………………………………………………………………………..

Detail any serious illness or operations:

…………………………………………………………………………………………………………………...

Detail any medication you take regularly (e.g. painkillers, anti-inflamatory drugs, oral contraceptives, HRT):

………………………………………………………………………………………………………………………………

If applicable, is there any possibility you may be pregnant?

Would you be able to travel to the Rowett Research Institute?

Would you be available to take part? Give details of when you might not be available:

Are you a blood donor, if so when did your last donate?

Would you object to your name being put onto our volunteer database **🔾** Yes **🔾** No

Please include any other information you may consider relevant:

………………………………………………………………………………………………………………….

Date: …………………………

**Please return to:**

Lynn Crosbie

Human Trials Coordinator

Provexis plc

c/o The University of Aberdeen Rowett Institute of Nutrition and Health

Greenburn Road, Aberdeen, AB21 9SB.

Tel: (01224) 715 753 E-mail: lynn.crosbie@provexis.com

**SUBJECT NUMBER:**
