# Supplementary material for: A randomised controlled trial comparing a dietary antiplatelet, the water-soluble tomato extract Fruitflow, with 75 mg aspirin in healthy subjects
Source: Eur J Clin Nutr. 2016 Nov 23;71(6):723–30. doi: 10.1038/ejcn.2016.222 (PMC5470100; doi:10.1038/ejcn.2016.222)
Supplement: Supplementary Information_study screening part 4 [file ejcn2016222x4.doc]

*This study is funded by Provexis Natural Products Limited (**www.provexis.com), and* *run in association with The University of Aberdeen Rowett Institute of Nutrition and Health*

**Dietary Questionnaire**

| **SUBJECT NUMBER** |  |
| --- | --- |
| **DATE** |  |

To help us gain further information for the study, we would like to ask you to fill out this questionnaire regarding your usual consumption of certain food items. Please take as much time as you need and answer as accurately and honestly as you can.

| **DIETARY QUESTIONNAIRE:** | |
| --- | --- |
| How many pieces/portions of fruit do you eat in an average week? |  |
| How many pieces/portions of vegetables do you eat in an average week? |  |

| **FOOD FREQUENCY QUESTIONNAIRE:** | | |
| --- | --- | --- |
| **Keeping the past 4 weeks in mind, how often do you eat the following foods, and in what amount do you usually consume them:** | | |
|  | **Portion size** | **Frequency**  **(per week)** |
| Apples, fresh | One medium size, ca. 100 g |  |
| Apple Juice | Glass of 160 ml |  |
| Grapefruit, fresh | One, medium size, flesh only, ca. 160 g |  |
| Grapefruit Juice | Glass of 160 ml |  |
| Grapes, fresh | Small bunch, ca. 100 g |  |
| Grape juice (not wine) | Glass of 160 ml |  |
| Melon, fresh | Slice without skin, ca. 200 g |  |
| Orange Juice | Glass of 160 ml |  |
| Oranges, fresh | One medium size without skin, ca. 160g |  |
| Satsumas / Clementines | One without skin, ca. 60 g |  |
| Strawberries, fresh | Portion, ca. 100 g |  |
| Tomatoes, raw | One medium size, ca. 85 g |  |
| Tomato juice | Glass of 160 ml |  |
| Canned tomatoes | Small tin |  |
| Tomato puree | 2 tablespoons |  |
| Tomato soup | ½ tin |  |
| Tomato Ketchup/ relish/ chutney | 2 tablespoons |  |
| Pizza | Average meal portion |  |
| Chilled/frozen meals containing tomato | Meal for 1 |  |
| Pasta based meals containing tomato | Meal for 1 |  |
| Onions | ½ medium size |  |
| Garlic | 1 clove |  |
| Spicy Food | Meal for 1 |  |
| Oily fish | Average portion size |  |

**Please return to:**

Lynn Crosbie

Human Trials Coordinator

Provexis plc

c/o The University of Aberdeen Rowett Institute of Nutrition and Health

Greenburn Road

Aberdeen

AB21 9SB

Tel: (01224) 715753 Email: lynn.crosbie@provexis.com
